# Relatlimab: a novel drug targeting immune checkpoint LAG-3 in melanoma therapy

**DOI:** 10.3389/fphar.2023.1349081

**Published:** 2024-01-10

**Authors:** Jingjing Su, Yiting Fu, Zitong Cui, Zain Abidin, Jingsong Yuan, Xinmiao Zhang, Runmin Li, Chunzhen Zhao

**Affiliations:** ^1^ Key Laboratory of Molecular Pharmacology and Translational Medicine and Department of Pharmacology, College of Pharmacy, Weifang Medical University, Weifang, China; ^2^ Department of Pharmaceutical Sciences, College of Pharmacy and Health Sciences, St. John’s University, Queens, NY, United States

**Keywords:** relatlimab, LAG-3 inhibitor, nivolumab, metastatic melanoma, immune checkpoint

## Abstract

Relatlimab is a type of human immunoglobulin G4 monoclonal blocking antibody. It is the world’s first Lymphocyte-Activation Gene-3 (LAG-3) inhibitor and the third immune checkpoint inhibitor with clinical application, following PD-1 and CTLA-4. Relatlimab can bind to the LAG-3 receptor which blocks the interaction between LAG-3 and its ligand to reduce LAG-3 pathway-mediated immunosuppression and promote T-cell proliferation, inducing tumor cell death. On 18 March 2022, the U.S. FDA approved the fixed-dose combination of relatlimab developed by Bristol Myers Squibb with nivolumab, under the brand name Opdualag for the treatment of unresectable or metastatic melanoma in adult and pediatric patients aged 12 and older. This study comprehensively describes the mechanism of action and clinical trials of relatlimab and a brief overview of immune checkpoint drugs currently used for the treatment of melanoma.

## 1 Introduction

Melanoma is a type of malignant tumor that has seen an increasing incidence rate in recent years. It is primarily caused by a combination of genetic susceptibility and environmental exposure to factors such as ultraviolet (UV) radiation (Dzwierzynski, 2021; Rashid et al., 2023). Lighter skin, freckles, and blue-green eyes are characteristics that have been associated with a higher susceptibility to melanoma (Ahmed et al., 2020; Dzwierzynski, 2021). In some studies, it has been observed that there are more reported cases of melanoma in males compared to females (Quintanilla-Dieck and Bichakjian, 2019; Watts et al., 2021). Malignant melanoma has a high propensity for metastasis, with common sites of metastasis being lymph nodes, the skin, lungs, liver, and bones. The main treatment methods for malignant melanoma include surgical removal, immunotherapy, gene therapy, and biotherapy (also known as targeted therapy) (Ahmed et al., 2020). The primary treatment method for early-stage melanoma is surgical removal, currently, combination therapies involving immunotherapy, targeted therapy, and chemotherapy are being tested (Rashid et al., 2023). In addition, immune checkpoint inhibitors have shown significant efficacy in the treatment of advanced melanoma (Callahan et al., 2016; Pavri et al., 2016; Teixido et al., 2021). Therefore, there is an urgent need to research and develop effective immune checkpoint inhibitors for the treatment of melanoma.

The main characteristics of melanoma include its metastatic and invasive nature, the ability to evade cell apoptosis, unlimited replicative potential, and its resistance to growth inhibitors (Lugović-Mihić et al., 2019). Metastatic melanoma is a disease that has spread beyond the regional lymph nodes and compared to other types of melanoma, has a poor prognosis (Frankel et al., 2003). In recent years, immunotherapy has made significant progress in the treatment of melanoma and other cancers (Herzberg and Fisher, 2016). Prior to 2022, the main targets of immune checkpoint blockade included programmed cell death protein 1 (PD-1) and cytotoxic T-lymphocyte-associated antigen 4 (CTLA-4) (Herzberg and Fisher, 2016; Lazaroff and Bolotin, 2023). On 18 March 2022, the U.S. FDA approved the fixed-dose combination of relatlimab and nivolumab for the treatment of unresectable or metastatic melanoma in adult and pediatric patients aged 12 years and older. Relatlimab targets Lymphocyte-Activation Gene-3 (LAG-3) and its approval marked the first LAG-3 immune therapy to be approved globally. It is the third immune checkpoint inhibitor to be applied in clinical practice, following PD-1 and CTLA-4 (Opdualag, 2023b; Kaplon et al., 2023; Paik, 2022). The approval of the fixed-dose combination of relatlimab and nivolumab also signifies the emergence of the “strongest ever” dual immunotherapy for cancer.

We comprehensively discuss the mechanisms of relatlimab, as well as clinical trials assessing its safety and efficacy. And, we also provide an overview of current immune checkpoint drugs used in the treatment of melanoma.

## 2 Research advancements in the traditional treatment approaches for melanoma

The typical treatment methods for malignant melanoma include surgical resection, radiation therapy, chemotherapy, targeted therapy, and immunotherapy (Ahmed et al., 2020). Surgical excision is the cornerstone of treatment for stage II and III melanoma patients (van Zeijl et al., 2017). When the tumor area is large and cannot be alleviated through non-surgical or immunotherapy methods, debulking surgery is a viable option (McLoughlin et al., 2007). But simple surgical excision alone, although it can achieve local control of melanoma, it does not significantly improve survival rates (van Zeijl et al., 2017). The method of treating melanoma patients with radiation doses ranging from approximately 25 cGy–230 cGy, preferably between 100 cGy and 200 cGy, at least twice a day, was granted authorization in the United States in 2019 (Anderson, 2019). Radiation therapy can be used as adjuvant treatment to surgery in cases where surgical resection is not feasible. Radiation therapy typically has good tolerability, but it can have long-term effects on the skin and other tissues (Dabestani et al., 2021). The combination of radiation therapy and immunotherapy is currently undergoing active evaluation and holds promise as a treatment approach for tumors like melanoma (Shi, 2015). Chemotherapy is an adjuvant treatment method for melanoma. As a palliative agent for the treatment of melanoma, only Dacarbazine has been approved by the US FDA for the treatment of metastatic melanoma (Lee et al., 1995; Lo and Fisher, 2014; Chen et al., 2023). However, melanoma exhibits high resistance to conventional chemotherapy drugs, and adjuvant chemotherapy alone may not alleviate the symptoms in patients (Pavlick, 2002; Wilson and Schuchter, 2016).

Compared to traditional treatment methods for melanoma, targeted therapy and immunotherapy have shown improved prognosis for metastatic melanoma (Li et al., 2022; Lazaroff and Bolotin, 2023). BRAF V600, NRAS, NF1, and c-KIT are genetic mutations that contribute to the development of melanoma (LoRusso et al., 2020; Li et al., 2022). Among them, the mutated form of BRAF has attracted considerable attention due to its widespread occurrence and potential clinical targeting (LoRusso et al., 2020). The mutated BRAF variant activates the constitutive activity of the BRAF kinase, leading to excessive activation of the MAPK signaling pathway. Drugs such as vemurafenib can inhibit the mutated BRAF and demonstrate significant survival (LoRusso et al., 2020). There are also patents that indicate a positive effect of combining BRAF inhibitors with anti-microtubule compounds or microtubule inhibitors for the treatment of metastatic melanoma (Wang et al., 2020). However, targeted therapies such as BRAF inhibitors, while able to quickly control symptoms, may lead to drug resistance and disease progression as they can reactivate MAPK signaling (Sun et al., 2020; Lazaroff and Bolotin, 2023).

Traditional immune therapies for melanoma include interferon (IFN), *Bacillus* Calmette-Guérin (BCG) vaccine, and high-dose interleukin-2 (IL-2), which have been shown to produce low but sustained response rates (Morton et al., 1974; Di Trolio et al., 2014). Compared to traditional immunotherapies, immune checkpoint inhibitors are the first drugs to demonstrate improved survival rates in patients with advanced melanoma. Common immune checkpoint inhibitors include previously marketed CTLA-4 inhibitors and PD-1 inhibitors, as well as newly marketed LAG-3 inhibitor drugs (Ralli et al., 2020; Rashid et al., 2023).

## 3 Mechanism of action and related drugs of CTLA-4 inhibitor and PD-1 inhibitor for the treatment of melanoma

**FIGURE 1:**
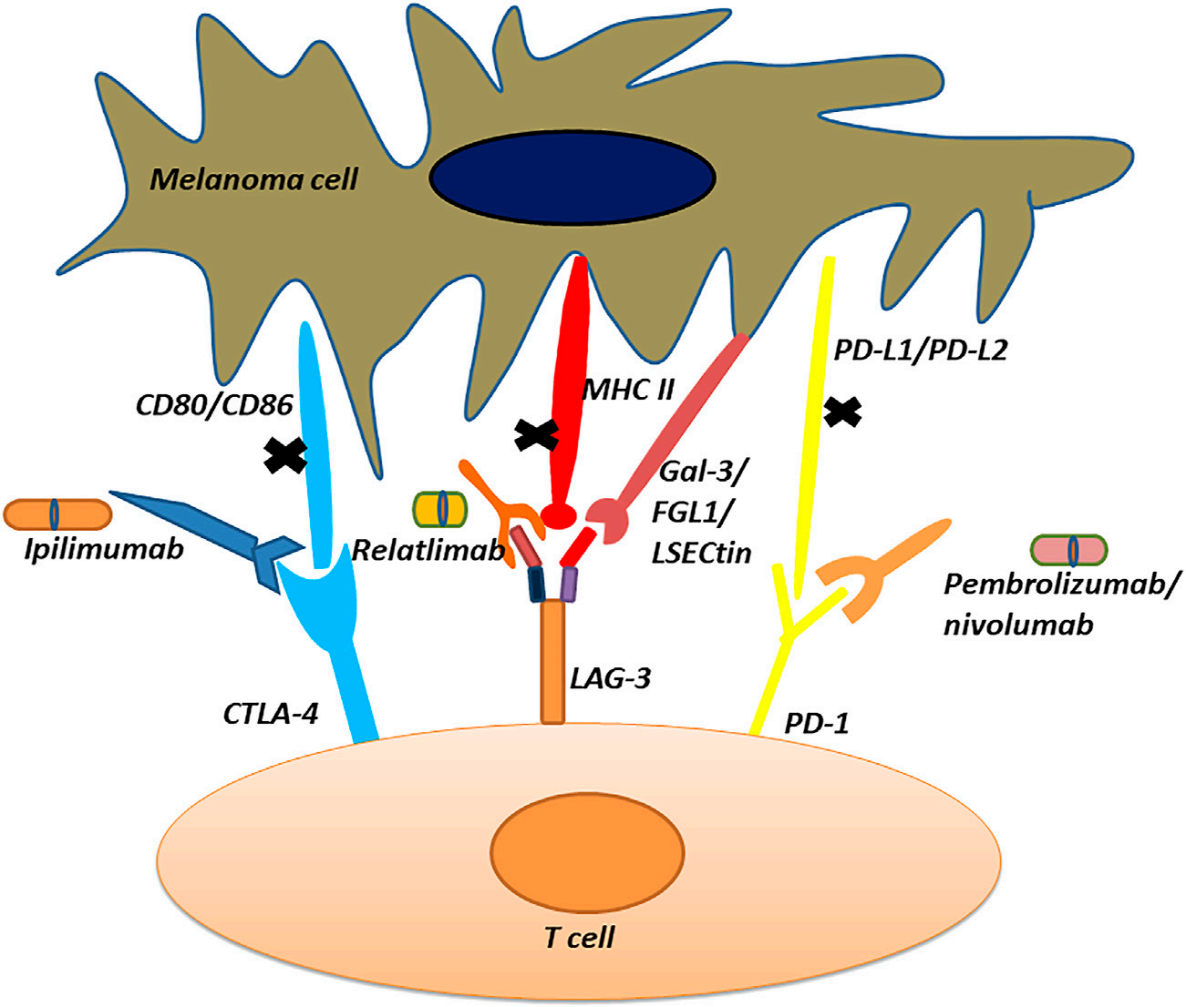
Mechanism of action of immune checkpoint inhibitors.

CTLA-4, also known as CD152, is primarily present on the surface of activated T-cells (Robert and Mateus, 2011). As an immune checkpoint protein, it plays a negative regulatory role in immune responses (Buchbinder and Desai, 2016). When stimulated by antigens, CTLA-4 is expressed on the surface of T-cells and binds to its ligands: CD80 or CD86, thereby, inhibiting self-activation and proliferation (Chikuma, 2017). Certain tumor cells can also express CD80 or CD86, and when bound to CTLA-4 can inhibit immune responses and promote tumor growth. Based on this characteristic of tumor cells, when CTLA-4 inhibitors are used, the binding of CTLA-4 to its ligands is blocked to cause T-cell activation and promote immune responses, thus suppressing the growth of tumor cells (Masteller et al., 2000). This effect of CTLA-4 inhibitors has a positive impact on the treatment of cancers such as melanoma (Snyder et al., 2014; Marshall and Djamgoz, 2018). The mechanism of action of CTLA-4 immune checkpoint inhibitors is illustrated in Figure 1. Ipilimumab, marketed as Yervoy, is the first CTLA-4 inhibitor approved for the treatment of metastatic melanoma (Fellner, 2011; Ledford, 2011). While it has shown some efficacy in treating melanoma, this drug only extends patients’ lives by a little over 4 months on average (Fellner, 2011). In most clinical trials, both as monotherapy or in combination therapy, Ipilimumab has shown immune-mediated adverse reactions (Fellner, 2011; Cameron et al., 2011). The most common severe immune-related adverse events include enterocolitis, hepatitis, dermatitis, neuropathy, and endocrinopathy (Cameron et al., 2011; Fellner, 2011; Thielsen and Svane, 2015).

PD-1 is a protein belonging to the immune checkpoint family, primarily found on the surface of activated immune cells such as T-cells, B-cells, and natural killer cells (Seidel et al., 2018). By binding with its ligands, PD-L1 or PD-L2, it inhibits the activation and function of T-cells, helping to maintain immune system balance. However, tumor cells can exploit this mechanism to evade immune attack and promote their own growth. By using PD-1 or PD-L1 inhibitors, the binding of PD-1 with its ligands can be blocked, leading to the activation of T-cells and enhancing their ability to kill tumor cells (Seidel et al., 2018; Dermani et al., 2019; Jiang et al., 2019). This immunotherapy targeting PD-1 has shown significant efficacy in various cancer types including melanoma and lung cancer (Dermani et al., 2019; Carlino et al., 2021). The mechanism of action of PD-1 immune checkpoint inhibitors is shown in Figure 1.

Pembrolizumab and nivolumab are PD-1 inhibitors that are currently available on the market for the treatment of melanoma (Poole, 2014; Raedler, 2015). Pembrolizumab, a humanized monoclonal anti-PD-1 antibody, developed by Merck & Co, is used in patients who have experienced disease progression after prior treatment with ipilimumab, as well as in patients with BRAF V600 mutation-positive melanoma as a BRAF inhibitor (Poole, 2014). Multiple phase III clinical trials have demonstrated that pembrolizumab can significantly prolong patients’ survival without causing a significant decrease in health-related quality of life (HRQOL) (Bottomley et al., 2021; Eggermont et al., 2021; Luke et al., 2022). However, in patients receiving pembrolizumab (including lymphoma and other solid tumors), over 60% of patients experienced adverse reactions, including thyroid dysfunction, hepatitis, and pneumonitis, with hepatitis and pneumonitis being more severe (Kwok et al., 2016; Robert et al., 2021). On 22 December 2014, the FDA approved nivolumab (Opdivo), developed by Bristol-Myers Squibb, for the treatment of patients with unresectable or metastatic melanoma and disease progression following treatment with Ipilimumab. This marked the emergence of the second PD-1 inhibitor approved for unresectable or metastatic melanoma (Raedler, 2015). Consistent with the anticipated outcomes, nivolumab is better tolerated than ipilimumab and has long-term treatment advantages (Ascierto et al., 2020). In addition, clinical trials have demonstrated that nivolumab monotherapy or nivolumab plus ipilimumab combination therapy also has positive effects on patients with metastatic uveal melanoma or active melanoma brain metastases (Long et al., 2018; Pelster et al., 2021). Meanwhile, the patent containing the administration of a combination of nivolumab and ipilimumab in a ratio of 1:3 was granted in Europe in 2020 (SADINENI et al., 2020). However, similar to ipilimumab monotherapy, both nivolumab monotherapy (29%) and nivolumab plus ipilimumab combination therapy (71%) had a high incidence of grade 3-4 treatment-related adverse events (Livingstone et al., 2022).

Thus, we see that when PD-1 and CTLA-4 inhibitors are used in combination, they have the highest durable response rate and remain effective in various situations, including brain metastases. However, up to 60% of patients experience multiple life-threatening toxicities (Amaria et al., 2018; Kreidieh and Tawbi, 2023). As LAG-3 is an immune checkpoint that associated with the activity of T cells, research has shown that LAG-3 is upregulated in tumor-infiltrating lymphocytes in several types of tumors, including melanoma (Workman et al., 2004; Grosso et al., 2007; Hemon et al., 2011; Durham et al., 2014). Therefore, the development of effective LAG-3 inhibitors holds promise as a new direction for melanoma treatment.

## 4 Relatlimab, the first LAG-3 inhibitor for metastatic melanoma

### 4.1 Mechanisms of action of relatlimab

LAG-3 is an inhibitory immune checkpoint protein expressed on the surface of certain T-cells (such as CD4^+^ T cells, CD8^+^ T cells, regulatory T-cells (treg) and natural killer cells (NK)). When T-cells are activated, the expression of LAG-3 becomes more prominent. LAG-3 is composed of 498 amino acids and its encoding gene is located on the distal short arm of chromosome 12, adjacent and homologous to CD4. LAG-3 consists of four extracellular immunoglobulin superfamily (Ig) domains (D1-D4). The ligands of it include Major Histocompatibility Complex Class II (MHC II), Galectin-3 (Gal-3), Fibrinogen-like protein 1 (FGL1), and Liver Sinusoidal Endothelial Cell Lectin (LSECtin). MHC II is the typical ligand, which can bind to the D1 domain of LAG-3 and inhibit T-cell activation and function. In the tumor microenvironment, MHC-II can present peptides on antigen-presenting cells (APCs) (including dendritic cells, macrophages, or B cells) to be recognized by CD4^+^ T-cells, leading to the activation of CD4^+^ T-cells. APCs deliver tumor antigens to naïve T-cells via MHC II, and then MHC-II binds to the T-cell receptor (TCR) and CD4, inducing T-cell activation. The activation of T-cells leads to an increased expression of LAG-3. LAG-3 binds to MHC-II, thereby inhibiting the interaction between MHC-II, TCR, and T-cells. As a result, the transmission of TCR signals is suppressed, leading to the inhibition of T-cell activation. This ultimately hinders the immune system’s ability to eliminate tumor cells (Graydon et al., 2020; Shi et al., 2021; Kreidieh and Tawbi, 2023; van Akkooi, 2023; Ziogas et al., 2023).

In March 2022, the U.S. FDA approved the fixed-dose combination of relatlimab and nivolumab for the treatment of unresectable or metastatic melanoma in adult patients and pediatric patients aged ≥12 years and weighing ≥40 kg (Paik, 2022). Relatlimab, the first LAG-3 inhibitor globally, is the third immune checkpoint inhibitor to be used in clinical practice following PD-1 and CTLA-4 (FDA, 2022; Opdualag, 2023b; Paik, 2022). It can bind to LAG-3 and block its ability to bind with MHC-II ligands. This inhibition leads to the suppression of LAG-3’s inhibitory effect on T-cells, thereby restoring T-cell immune activity and enhancing the ability of T-cells to kill melanoma cells (Thudium et al., 2022; van Akkooi, 2023). The mechanism of action of relatlimab is illustrated in Figure 1. And the dosage used is as follows. The recommended dose for the fixed-dose combination of relatlimab and nivolumab (Opdualag, 2023b) is 160 mg of relatlimab plus 480 mg of nivolumab for adults weighing more than 40 kg. It should be administered intravenously every 4 weeks (maximum dose > 160 mL), with a maximum dose of 4 mL/kg for adults weighing less than 40 kg. For children 12 years of age or older and younger than 18 years old, dose reduction is not recommended. At a dose of 160 mg every 4 weeks, relatlimab exhibits nonlinear and time-varying pharmacokinetics in patients with different cancers. When relatlimab and nivolumab combined medication, the steady-state, geometric mean maximum and mean plasma concentrations of relatlimab were 62.2 μg/mL and 28.8 μg/mL, respectively, while those of nivolumab were 187 μg/mL and 94.4 μg/mL, respectively (Paik, 2022). Additionally, relatlimab can also impact the functionality of immune cells like natural killer (NK) cells, promoting their activity and enhancing their ability to recognize and attack melanoma cells (Thudium et al., 2022; van Akkooi, 2023). To follow, we provide a detailed overview of the preclinical and clinical studies of relatlimab, and briefly introduce the combination of LAG-3, PD-1 and CTLA-4 related immune checkpoint inhibitors.

### 4.2 Treatment and preclinical studies related to relatlimab

High expression of LAG-3 has been observed in various tumor diseases such as neuroendocrine tumors, uterine cancer, colorectal cancer, melanoma, and others (Adashek et al., 2023). While relatlimab was initially approved for the treatment of metastatic melanoma, its potential positive effects on other diseases have been reported earlier. Research has shown that LAG-3 expression is significantly dysregulated on leukemia cells, NK cells, and T-cells, which can contribute to the development of chronic lymphocytic leukemia (CLL) disease (Sordo-Bahamonde et al., 2021). After treatment with relatlimab, the LAG-3 signaling pathway is markedly inhibited, enhancing the anti-tumor response mediated by NK cells and T-cells, thus exerting a positive effect on the treatment of CLL disease (Sordo-Bahamonde et al., 2021). Nivolumab, a PD-1 inhibitor, has been approved for second-line treatment of advanced HCC. Relatlimab and nivolumab can individually inhibit LAG-3 and PD-1, respectively, thereby activating T-cells and improving immune response. A randomized, open-label phase II clinical trial studying the use of relatlimab and nivolumab in treating advanced hepatocellular carcinoma patients who have progressed on tyrosine kinase inhibitor therapy is currently recruiting volunteers (Sangro et al., 2021). Similarly, relatlimab is being investigated in phase II clinical trials for the treatment of renal cell carcinoma and advanced renal medullary carcinoma (Lecocq et al., 2020; Schoenfeld et al., 2022). Additionally, clinical trials have demonstrated that LAG-3 inhibitors have shown promising therapeutic effects in diffuse large B-cell lymphoma, TNBC (advanced triple-negative breast cancer), and GYNC (gynecologic cancers) (Carey et al., 2021; Li et al., 2023).

Immune checkpoint inhibitors represent a novel approach in the treatment of oncologic diseases such as melanoma. The use of PD-1 inhibitors, CTLA-4 inhibitors, or their combination has shown certain therapeutic effects in melanoma treatment. However, over 60% of patients experience severe or life-threatening toxic reactions (Fellner, 2011; Cameron et al., 2011; Curry et al., 2017; Kreidieh and Tawbi, 2023; Ledford, 2011; O'Day et al., 2010; Ribas et al., 2013; Thielsen and Svane, 2015; Wolchok et al., 2010). Particularly, there is a positive correlation between the incidence of skin toxicity and favorable outcomes such as progression-free survival, tumor response, and overall survival in patients (Freeman-Keller et al., 2016; Hua et al., 2016). Preclinical experiments have shown that LAG-3 and PD-1 have a synergistic effect in inhibiting T-cell activation, thus promoting tumor immune evasion (Okazaki et al., 2011; Zelba et al., 2019). In mice, simultaneous blockade of both receptors has shown a more potent immune response compared to blocking either receptor alone (Woo et al., 2012). Similar findings were observed in human *in vitro* experiments with melanoma/T-cell co-cultures, indicating that simultaneous blockade of PD-1 enhance T-cell response and controls tumor cell growth, in contrast to blocking LAG-3 alone (Gestermann et al., 2020). In toxicity studies on cynomolgus monkeys, the combined use of LAG-3 and PD-1 inhibitors (i.e., coadministration of relatlimab and nivolumab) has demonstrated good tolerability (Thudium et al., 2022). This suggests that the combination therapy of relatlimab and nivolumab is a well-tolerated regimen for patients with unresectable or metastatic melanoma (Thudium et al., 2022).

### 4.3 Phase I/IIa clinical trial (the safety and efficacy comparison of the combination of relatlimab with nivolumab)

RELATIVITY-020 is a phase I/IIa dose escalation and cohort expansion study, and a cohort study in Part C evaluated the safety and efficacy of the combination of relatlimab and nivolumab. In this study, 68 patients were enrolled, with 57% of patients having prior experience with CTLA-4 inhibitors and 46% having received third-line therapy. All patients received 80 mg of relatlimab and 240 mg of nivolumab intravenously every 2 weeks. The primary endpoints were the safety of the drugs and objective response rate (ORR), disease control rate (DCR), and duration of response (DOR). Results showed that among the 61 evaluable patients, 1 patient achieved complete response, 6 patients achieved partial response, resulting in an ORR of 11.5%. The DCR was 49% but did not reach the median DOR. When comparing patients with LAG-3 expression <1% to those with LAG-3 expression ≥1%, the ORR was approximately 3.5 times higher in the latter group, while PD-L1 expression did not seem to impact ORR. The incidence of treatment-related adverse events in this trial was 51%. The trial demonstrated that patients tolerated relatlimab and the combination of relatlimab and nivolumab (anti-LAG-3 + anti-PD-1 therapy) well. Additionally, these treatment approaches showed clinically meaningful anti-tumor activity. In terms of safety, the combination had a similar profile to nivolumab alone and patient response rates were associated with LAG-3 expression but not with PD-L1 expression (Ascierto et al., 2017a; Ascierto et al., 2017b).

In Part D of the RELATIVITY-020 trial, Ascierto’s team conducted a study in 518 patients (In the previous use of PD-L1 inhibitors, there is documented disease progression) with unresectable melanoma who were divided into two groups: the D1 group (354 patients) had received prior treatment with one PD-L1 inhibitor, while the D2 group (164 patients) had received prior treatment with more than one PD-L1 inhibitor. The aim of the trial was to confirm the safety of using relatlimab alone and in combination with nivolumab, as well as to observe the ORR and progression-free survival (PFS) with both drugs. In the D1 portion, patients were required to receive relatlimab + nivolumab at a dose of 80/240 mg (189 patients) administered every 2 weeks (SAV), or at a dose of 160/480 mg in a randomized 1:1 manner, administered every 4 weeks (FDC—82 patients and SAV—83 patients). In the D2 portion, 164 patients received relatlimab + nivolumab treatment at a dose of 160/480 mg every 4 weeks (SAV). In the SAV group, the median duration of relatlimab + nivolumab treatment was 16 weeks, while in the FDC group it was approximately 19.8 weeks. In the overall evaluable population of this clinical trial, the ORR for patients in the D1 group was 12.0%, while it was 9.2% for the D2 group. The DCR was 40.5% for the D1 group and 39.9% for the D2 group. In the D1 group, when patients had a ≥1% expression of LAG-3, the ORR was 14.1%, whereas it was 5.4% when LAG-3 expression was <1%. When patients had a ≥1% expression of PD-L1, the ORR was 15.7%, compared to 8.2% when PD-L1 expression was <1%. The ORR was 11.7% when CTLA-4 was expressed in patients, and 12.1% when it was not expressed. The ORR was 13.5% for patients who had previously received BRAF/MEK inhibitors, and 12.5% for those who had not received BRAF/MEK inhibitors. In the D1 group, the median DOR was not reached, while in the D2 group, the DOR was 12.8 months. In the D1 group, the percentage of responders who maintained their response at 6 months was 92.3%, compared to 84.6% in the D2 group. For those who maintained response at 12 months, the percentage was 70.9% in the D1 group and 52.7% in the D2 group. In the D1 group, the median PFS was 2.1 months, with a PFS rate of 29.1% at 6 months and 21.4% at 12 months. In the D2 group, the median PFS was 3.2 months, with a PFS rate of 27.7% at 6 months and 16.0% at 12 months. In this clinical trial, in the D1 group, the median overall survival (OS) was 14.7 months, with a 12-month OS rate of 56.0%. In the D2 group, the median OS was 17.1 months, with a 12-month OS rate of 60.0% (Ascierto et al., 2023).

In Part D of the RELATIVITY-020 trial, two administration methods, SAV and FDC were used for relatlimab + nivolumab at doses of 160/480 mg, administered every 4 weeks. It was observed that the levels of soluble LAG-3 decreased by 50% in patients using both the SAV and FDC administration methods. The impact of the two administration methods on LAG-3 levels was comparable, indicating that the co-administration of the two agents as an FDC formulation did not have an impact on pharmacokinetics. The safety investigation of this clinical trial showed that in the D1 group, the incidence of treatment-related adverse events (TRAEs) of any grade was 67.5%, with a 15% incidence of grade 3-4 TRAEs. The most common immune-mediated adverse events were rash, hypothyroidism/thyroiditis, and diarrhea/colitis. In the D2 group, the TRAE incidence was 68.9%, with a 12.8% incidence of grade 3-4 TRAEs. The most common immune-mediated adverse events were rash, hypothyroidism/thyroiditis, and hepatitis (Ascierto et al., 2023).

The RELATIVITY-020 trial demonstrated that the combination therapy of relatlimab and nivolumab is safe and provides durable clinical benefits for patients with advanced melanoma who have previously received PD-L1 inhibitors (Ascierto et al., 2023).

### 4.4 Phase II/III clinical trial (the comparison of efficacy between the combination of relatlimab with nivolumab and nivolumab monotherapy)

RELATIVITY-047 is a global, randomized, double-blind, Phase II/III study. A total of 714 patients were randomly selected for this study, with 355 patients being allocated in a 1:1 ratio to receive the combination therapy of relatlimab (160 mg) and nivolumab (480 mg) (FDC), and 359 patients receiving nivolumab (480 mg) monotherapy. All patients received intravenous injections every 4 weeks. The primary objective of this trial is to compare the difference in PFS between the combination therapy of relatlimab and nivolumab versus nivolumab monotherapy. Additionally, the study also aims to compare the differences in OS and ORR. In this clinical trial, the median follow-up duration was 19.3 months. The median PFS for the combination therapy of relatlimab and nivolumab was 10.2 months (95% CI, 6.5–14.8), compared to 4.6 months (95% CI, 3.5–6.4) for nivolumab monotherapy (HR 0.78; 95% CI, 0.6–0.9). The combination therapy of relatlimab and nivolumab did not reach the median OS (NR; 95% CI, 34.2-NR), while the median OS for nivolumab monotherapy was 34.1 months (95% CI, 25.2-NR) (HR 0.80; 95% CI, 0.6–1.0; *p* = 0.0593). The 12-month OS rate for the combination therapy of relatlimab and nivolumab was 77.0% (95% CI, 72.2–81.1), and the 24-month OS rate was 63.7% (95% CI, 58.1–68.7). The 12-month OS rate for nivolumab monotherapy was 71.6% (95% CI, 66.6–76.0), and the 24-month OS rate was 58.3% (95% CI, 52.7–63.4). The ORR for the combination therapy of relatlimab and nivolumab was 43.1% (95% CI, 37.9–48.4), with a complete response rate of 16.3%. The ORR for nivolumab monotherapy was 32.6% (95% CI, 27.8–37.7), with a complete response rate of 14.2% (Lipson et al., 2018; Hodi et al., 2021; Lipson et al., 2021; Long et al., 2022; Tawbi et al., 2022; Raschi et al., 2023). As shown in Table 1.

**TABLE 1:** Comparison of the efficacy of combination therapy of relatlimab and nivolumab versus nivolumab monotherapy in the treatment of melanoma with a median follow-up time of 19.3 months.

	Relatlimab and nivolumab (*n* = 355)	Nivolumab (*n* = 359)
Median PFS (95% CI)	10.2 months (6.5–14.8)	4.6 months (3.5–6.4)
Median OS (95% CI)	NR (34.2–NR)	34.1 months (25.2–NR)
OS rate,% (95% CI)		
12 months	77.0 (72.2–81.1)	71.6 (66.6–76.0)
24 months	63.7 (58.1–68.7)	58.3 (52.7–63.4)
ORR, % (95% CI)	43.1 (37.9–48.4)	32.6 (27.8–37.7)

Although the combination therapy of relatlimab and nivolumab has demonstrated significant efficacy in terms of PFS, it is associated with a higher incidence of treatment-related adverse events (81.1% vs. 69.9%) compared to nivolumab monotherapy. Itchiness/rash, fatigue, joint pain, thyroid dysfunction/thyroiditis/thyroid hyperactivity, and diarrhea/colitis are the most common treatment-related adverse events and 5.9% of patients experienced infusion-related adverse reactions (3.6% with nivolumab monotherapy). In terms of safety, when relatlimab and nivolumab were used in combination, 75 cases (21.1%) of grade 3-4 treatment-related adverse events were observed, along with 54 cases (15.2%) of any-grade TRAEs leading to treatment discontinuation. Additionally, 4 cases resulted in fatalities. In the nivolumab monotherapy group, 40 cases (11.1%) of grade 3-4 TRAEs and 26 cases (7.2%) of any-grade TRAEs leading to treatment discontinuation were observed. There were 2 cases of fatalities. This phase II/III clinical trial demonstrates that the combination therapy of relatlimab and nivolumab has a more significant PFS, lower risk of death, and higher objective response rate compared to nivolumab monotherapy, with a safe and reliable safety profile. This suggests that the combination therapy of relatlimab and nivolumab has potential value in the treatment of metastatic melanoma (Lipson et al., 2018; Hodi et al., 2021; Lipson et al., 2021; Long et al., 2022; Tawbi et al., 2022; Raschi et al., 2023).

In another clinical trial, the authors described the impact of the combination therapy of relatlimab and nivolumab compared to nivolumab monotherapy on patient outcomes including PFS, OS, and ORR within predefined stratification factors such as LAG-3 expression, PD-L1 expression, BRAFV600 mutation status, and metastatic stage. Patients were randomized in a 1:1 ratio to receive either the combination therapy of relatlimab (160 mg) plus nivolumab (480 mg) in a fixed-dose combination (FDC) or nivolumab (480 mg) monotherapy, administered intravenously every 4 weeks at the same dosages as in the RELATIVITY-047 trial. The experimental results showed that the combination therapy of relatlimab and nivolumab had a higher PFS than nivolumab monotherapy in all patients. The trends in OS and ORR were also similar between the two groups. Specifically, when LAG-3 expression was ≥1%, the ORR in the group receiving the combination therapy of relatlimab and nivolumab was 47%, compared to 35% in the nivolumab monotherapy group; when LAG-3 expression was <1%, the ORR was 31% and 24%, respectively. Similarly, when PD-L1 expression was ≥1%, the ORR was 53% and 45%; and when PD-L1 expression was <1%, the ORR was 36% and 24%, respectively. In patients with BRAF wild-type melanoma, the ORR was 43% and 34% in the group receiving the combination therapy of relatlimab and nivolumab and the nivolumab monotherapy group, respectively. In patients with BRAF mutant melanoma, the ORR was 43% and 31%, respectively. In this trial, the combination therapy of relatlimab and nivolumab also demonstrated manageable safety. The results above indicate that in melanoma patients expressing different immune checkpoint proteins, the combination therapy of relatlimab and nivolumab showed superiority in terms of PFS, OS, and ORR compared to the nivolumab monotherapy group (Hussein et al., 2022).

Similar to RELATIVITY-047, in 2022, a clinical trial conducted by Rodabe N. Amaria’s team also yielded similar results. The trial involved 30 patients with resectable stage III or oligometastatic stage IV melanoma. The patients received two doses of combined treatment with relatlimab (160 mg) + nivolumab (480 mg) administered intravenously every 4 weeks, followed by surgery. Subsequently, they received 10 additional adjuvant combination therapy doses at the same dosage as before. The primary endpoint was the complete response rate (pCR) in patients. The results of the study showed that the pCR rate with combination therapy was 57%, with an overall pathologic response rate of 70%. The radiologic response rate was 57%, and no grade 3-4 immune-related adverse events were observed. The 1-year recurrence-free survival rates were 100% for patients with any pathologic response and 88% for those without, while the corresponding 2-year recurrence-free survival rates were 92% and 55%, respectively. This trial further demonstrates the safety and efficacy of combined treatment with relatlimab and nivolumab for melanoma (Amaria et al., 2022).

## 5 Results after 2 years of combination therapy with relatlimab and nivolumab, as well as the health-related quality of life

Similar to the RELATIVITY-047 clinical trial, patients were randomly assigned in a 1:1 ratio to receive either the combination therapy of relatlimab (160 mg) and nivolumab (480 mg) (FDC) or nivolumab (480 mg) monotherapy, administered intravenously every 4 weeks. However, unlike previous studies, the researchers conducted a longer follow-up period and reported relevant outcomes such as PFS, OS and ORR. The median follow-up time in this clinical trial was 25.3 months, with the shortest follow-up time being 21.0 months. Similar to previous reports, the combinational therapy of relatlimab and nivolumab showed superior PFS, OS, and ORR compared to the monotherapy of nivolumab, as shown in Table 2. For safety, 61 patients (17.2%) in the combinational therapy group and 31 patients (8.6%) in the nivolumab monotherapy group experienced TRAEs, respectively. Correspondingly, 78 patients (22.0%) and 43 patients (12.0%) had grades 3-4 TRAEs. There were a total of 6 treatment-related deaths (4 in the combinational therapy group and 2 in the nivolumab monotherapy group) (Hodi et al., 2022). The results of this clinical trial are consistent with previous findings, suggesting that the combinational therapy of relatlimab and nivolumab has better clinical efficacy than nivolumab monotherapy.

**TABLE 2:** Comparison of the efficacy of combination therapy of relatlimab and nivolumab versus nivolumab monotherapy in the treatment of melanoma with a median follow-up time of 25.3 months.

	Relatlimab and nivolumab (*n* = 355)	Nivolumab (*n* = 359)
Median PFS (95% CI)	10.2 months (6.5–14.8)	4.6 months (3.5–6.5)
Median OS (95% CI)	NR (31.5–NR)	33.2 months (25.2–45.8)
OS rate,%(95% CI)		
24 months	61.8 (56.5–66.6)	58.3 (52.9–63.2)
36 months	54.1 (48.6–59.3)	48.4 (42.9–53.8)
48 months	51.5 (45.9–56.9)	42.5 (36.4–48.5)
ORR, % (95% CI)	43.7 (38.4–49.0)	33.7 (28.8–38.9)
TRAEs	61 patients (17.2%)	31 patients (8.6%)
Grades 3-4 TRAEs	78 patients (22.0%)	43 patients (12.0%)

Health-related quality of life (HRQOL) refers to the impact of illness, medical interventions, aging, and changes in the social environment on individuals’ health status, as well as their subjective experiences related to their economic, cultural background, and values. In the phase II/III RELATIVITY-047 clinical trial, it has been demonstrated that relatlimab and nivolumab improve PFS in patients with metastatic melanoma (median follow-up of 13.2 months and 25.3 months). In this clinical study, researchers also observed the health status of previously treated melanoma patients. Patients were randomly assigned in a 1:1 ratio to receive combination therapy of relatlimab and nivolumab (160 mg and 480 mg, respectively, as a fixed-dose combination) or monotherapy with nivolumab (480 mg) alone. Both treatment regimens were administered intravenously every 4 weeks. The primary assessments included the FACT-M total score, FACT-M Trial Outcome Index, FACT-G total score, FACT-M Melanoma Subscale (MS), EQ-5D-3L Health Utility Index, and EQ-5D-3L Visual Analog Scale (EQ-VAS) (Schadendorf et al., 2023).

Among the 355 patients receiving combination therapy of relatlimab and nivolumab, 317 cases were available for FACT-M assessment, and 315 cases were available for EQ-5D-3L evaluation. Similarly, among the 359 patients using nivolumab alone, 327 cases were included in the FACT-M assessment, and 323 cases were included in the EQ-5D-3L evaluation. Clinical trial results indicate that in both the combination therapy group of relatlimab and nivolumab and the nivolumab monotherapy group, the proportions of patients showing significant improvement, no change, or significant deterioration in both FACT-M scores and EQ-5D-3L scores remained similar and stable during the treatment period. From week 96 to week 152 post-treatment, FACT-M scores showed that the proportion of patients experiencing deterioration was higher in the combination therapy group compared to the nivolumab monotherapy group. At the first follow-up, the proportion of patients experiencing worsening of symptoms after discontinuation of treatment increased in both groups and then decreased to treatment levels. For the FACT-G item GP5, at the 144-week treatment time point, the proportion of patients in both treatment groups who reported being “quite a bit” or “very much” bothered by TRAEs was <6%, these findings indicate that the overall tolerability of the combination therapy of relatlimab and nivolumab is similar to nivolumab monotherapy. Additionally, in both treatments, the average change in FACT-M total score and EQ-VAS score compared to baseline was mostly maintained within the average range. Responders had a better HRQOL than non-responders early in treatment for both groups. These findings suggest that the improvement of disease status during treatment may contribute to early improvements in HRQOL (Schadendorf et al., 2023).

In summary, the above clinical trial results suggest that dual inhibition of LAG-3 and PD-1 through combinational therapy with relatlimab and nivolumab holds promise as a first-line treatment option for patients with metastatic melanoma.

## 6 The safety associated with the combination use of relatlimab and nivolumab

Despite the significant PFS achieved with the combination use of relatlimab and nivolumab, treatment-related adverse reactions still exist. During or after treatment, relatlimab and nivolumab may inadvertently attack normal tissues and organs while killing cancer cells, which can potentially lead to severe, and in some cases, fatal outcomes. Furthermore, there are also some side effects that may occur. Common side effects include: pulmonary issues (such as cough), gastrointestinal problems (such as diarrhea), liver problems (such as bleeding tendencies), endocrine gland issues (such as increased sweating), renal problems (such as decreased urine output), skin problems (such as itching), cardiac issues (such as accelerated heart rate), neurological issues (such as changes in mood and behavior), visual issues (such as blurred vision), muscle problems (such as muscle spasms), and hematologic issues (such as decreased red blood cells). Importantly, this medication may increase the risk of transplant complications [such as graft-versus-host disease (GVHD)]. Therefore, patients who have received allogeneic bone marrow (stem cell) transplantation with donor cells may experience severe and potentially life-threatening adverse reactions if they undergo treatment with relatlimab and nivolumab again. So, during and after the use of relatlimab and nivolumab, patients should observe any changes in all aspects of their body in a timely manner. (https://www.opdualag.com/results/side-effectsv) (https://www.mayoclinic.org/drugs-supplements/nivolumab-relatlimab-rmbw-intravenous-route/side-effects/drg-20531825).

In order to minimize the related side effects and increase the safety of medication, patients should also pay attention to the following issues. During the treatment and within 5 months from the last administration of relatlimab and nivolumab, patients should take contraceptive measures. Additionally, it is currently unclear whether this combination has an impact on breast milk. Therefore, breastfeeding is not recommended during this period. The safety and efficacy of this combination have not been established in children aged 12 years and under, as well as in children aged 12 years and above with body weight less than 40 kg. There is currently insufficient evidence to suggest that the combined use of relatlimab and nivolumab has specific effects on elderly patients. This drug should be discontinued for patients with severe or life-threatening infusion-related reactions. (https://www.opdualag.com/results/side-effectsv) (https://www.mayoclinic.org/drugs-supplements/nivolumab-relatlimab-rmbw-intravenous-route/side-effects/drg-20531825).

## 7 Research progress of LAG-3 immune checkpoint drugs

Combination therapy with nivolumab and ipilimumab has shown promising results in the treatment of melanoma but is associated with more adverse reactions. Comparatively, the combination of relatlimab and nivolumab has demonstrated similar PFS but tends to exhibit earlier survival benefits and fewer TRAEs. This represents a significant advancement in the treatment of melanoma (Larkin et al., 2015; Larkin et al., 2019; Janjigian et al., 2021; Wolchok et al., 2022; Zhao et al., 2022). Currently, many new immune checkpoint drugs are undergoing clinical trials. The following summarizes the LAG-3 immune checkpoint drugs that are currently in clinical trials, excluding those for relatlimab (Chocarro et al., 2022; Li et al., 2023). As shown in Tables 3, 4.

**TABLE 3:** Clinical study of LAG-3 immune checkpoint drugs (monoclonal antibodies).

Drug	Intervention	Clinic trial	Phase	Indication
REGN3767 (Fianlimab)	REGN3767 + cemiplimab	NCT01042379	II	Breast carcinoma
Sym022	Sym021or	NCT03489369, NCT04641871, NCT03311412	I	Cancer
	Sym021 + Sym022or			
	Sym021 + Sym022 + Sym023			
GSK2831781 (IMP731)	—	NCT03965533, NCT02195349	I	Psoriasis
INCAGN02385	INCAGN02385	NCT03538028, NCT04370704	I	Advanced malignant neoplasm
	INCAGN02385+INCMGA0002+INCAGN02390		I/II	
TSR-033	—	NCT03250832	I	—
LAG525 (Ieramilimab)	LAG525 + spartalizumab	NCT02460224	I/II	Melanoma; renal cell carcinoma; mesothelioma
MK-4280 (Favezelimab)	MK-4280 or MK-4280 + pembrolizumab	NCT05064059	I/II	—

**TABLE 4:** Clinical study of LAG-3 immune checkpoint drugs (lg fusion proteins and bispecifics).

Drug	Intervention	Clinic trial	Phase	Indication
IMP321	IMP321 + avelumab	—	—	Advanced solid tumors

Drug	Target	Clinic trial	Phase	Indication
MGD013 (Tebotelimab)	PD-1 + LAG-3	—	I	Relapsed or refractory DLBCL
FS118	PD-1 + LAG-3	NCT03440437	I/II	Advanced malignant neoplasm
RO7247669	PD-1 + LAG-3	NCT04524871	—	—
		NCT04785820		
		NCT04140500		
EMB-02	PD-1 + LAG-3	NCT04618393	I/II	Advanced solid tumors
XmAb841 (Pavunalimab)	CTLA-4 + LAG-3	NCT03849469	I	Advanced solid tumors
IBI323	PD-1 + LAG-3	—	I	Advanced malignant neoplasm
CB213	PD-1 + LAG-3	—	—	—

## 8 Conclusion and future directions

Melanoma, as a highly prevalent disease, has numerous treatment options (Ahmed et al., 2020). Surgical treatment, radiotherapy, and chemotherapy are the traditional methods for treating melanoma but only provide limited benefits in terms of local disease control. And patients also exhibit some resistance to chemotherapy (Lee et al., 1995; Pavlick, 2002; McLoughlin et al., 2007; Lo and Fisher, 2014; Shi, 2015; Wilson and Schuchter, 2016; Dabestani et al., 2021; Chen et al., 2023). Targeted therapy and immunotherapy can improve the prognosis of metastatic melanoma (Li et al., 2022; Lazaroff and Bolotin, 2023). However, targeted therapy can activate the MAPK signaling pathway, leading to the development of resistance in melanoma and disease progression (Sun et al., 2020; Lazaroff and Bolotin, 2023). Currently, immune checkpoint inhibitors have become the main focus in the treatment of tumor diseases. CTLA-4 and PD-1 inhibitors were immune checkpoint drugs that preceded LAG-3 inhibitors (Ralli et al., 2020; Rashid et al., 2023). They have shown high rates of durable response and can prolong patients’ PFS, however, the use of these inhibitors, whether alone or in combination, is associated with relatively high toxicity (Amaria et al., 2018; Kreidieh and Tawbi, 2023).

Clinical studies have shown that the combination therapy of relatlimab and nivolumab significantly improves patients’ PFS (Hussein et al., 2022). The two-year survival rate of patients is significantly higher compared to previous immune checkpoint inhibitors, and they also demonstrate higher FACT-M and EQ-5D-3L scores (Hodi et al., 2022; Hussein et al., 2022; Schadendorf et al., 2023). This milestone marks the advent of the “strongest-ever” dual immune therapy. However, manageable adverse reactions still remain relatively high. It can potentially disrupt the functioning of organs and tissues such as the lungs, gastrointestinal tract, liver, kidneys, skin, heart, brain, muscles, and blood. Seriously, the combined use of relatlimab and nivolumab can lead to immune system attacking any normal organs and tissues in the body, thereby disrupting normal body functions. These adverse reactions can be particularly life-threatening for patients who have received allogeneic hematopoietic stem cell transplantation. Furthermore, existing clinical trials have only studied the 2-year survival outcomes of patients treated with relatlimab and nivolumab and effective data is still required to demonstrate the durable results of combination therapy with relatlimab and nivolumab (Thomas et al., 2023). Therefore, the use of relatlimab still faces significant challenges.

As the world’s first approved LAG-3 immune checkpoint inhibitor drug, relatlimab has brought significant survival benefits to patients with metastatic melanoma (Amaria et al., 2022; Hodi et al., 2022; Hussein et al., 2022; Schadendorf et al., 2023). However, there are still many unresolved issues in the research of LAG-3 immune checkpoint drugs. Firstly, more detailed research is needed on the specific mechanisms of how LAG-3 interacts with its ligands to negatively regulate T cell function. Secondly, besides MHC II, Gal-3, FGL1, and LSECtin (Graydon et al., 2020; Shi et al., 2021; Kreidieh and Tawbi, 2023; van Akkooi, 2023; Ziogas et al., 2023), there may be other potential ligands for LAG-3, which could provide clues for studying the mechanism of action of LAG-3 immune checkpoint inhibitor drugs. Thirdly, there may be variations in the clinical benefits of the same drug among patients with melanoma at different metastatic sites, and the development of treatment resistance may also occur. Lastly, the issue of combined use of multiple immune checkpoint drugs (toxicity, drug interactions, etc.,) remains a major challenge in cancer treatment.

Although relatlimab initially approved for the treatment of metastatic melanoma, there are ongoing active trials testing the approach of either selectively blocking LAG-3 or simultaneously blocking two or more immune checkpoints in other cancer diseases such as diffuse large B-cell lymphoma, triple-negative breast cancer (TNBC), gynecologic cancers, and leukemia (Carey et al., 2021; Sordo-Bahamonde et al., 2021; Li et al., 2023). Research has shown that relatlimab treatment reduces the number of leukemia cells by decreasing high levels of LAG-3 in the peripheral blood of patients with chronic lymphocytic leukemia, thereby promoting T cell production of cytokines and restoring anti-leukemia responses (Sordo-Bahamonde et al., 2021). In a mouse model of ovarian cancer, simultaneous blockade of LAG-3 and PD-1 promotes the production of cytokines by tumor antigen-specific CD8^+^ T cells, leading to a more effective therapeutic outcome against ovarian cancer (Matsuzaki et al., 2010; Huang et al., 2015). Among 363 patients with TNBC, 15% of patients exhibited co-expression of LAG-3 and PD-1 (Bottai et al., 2016). Research has shown that 27% of TNBC tissues exhibit PD-1^+^ and LAG-3^+^ cells. For patients with poor response to anti-PD-1 monotherapy, the dual blockade of PD-1 and LAG-3 may be a viable treatment option for TNBC (Wu et al., 2021). Additionally, there are numerous dual-specificity immune checkpoint inhibitors and fusion proteins in different stages of clinical trials for LAG-3 which will greatly advance the treatment of cancerous diseases (The relevant content is outlined in Table 4).

Immune checkpoint inhibitors have become key drugs in cancer treatment and the combination of multiple immune checkpoint inhibitors has become a hot topic in the treatment of tumor diseases. This article mainly introduces the clinical trial status of LAG-3 inhibitor and PD-1 inhibitor in the treatment of metastatic melanoma. Furthermore, we presented the adverse reactions of relatlimab, summarized immune checkpoint inhibitors currently in clinical development, and compared traditional treatment methods for melanoma with immune checkpoint inhibitor therapy. This may potentially provide new leads for the treatment of tumor diseases such as metastatic melanoma and the development of immune checkpoint inhibitors.
